# Of Black Swans and Tossed Coins: Is the Description-Experience Gap in Risky Choice Limited to Rare Events?

**DOI:** 10.1371/journal.pone.0020262

**Published:** 2011-06-01

**Authors:** Elliot A. Ludvig, Marcia L. Spetch

**Affiliations:** 1 Princeton Neuroscience Institute, Princeton University, Princeton, New Jersey, United States of America; 2 Department of Mechanical and Aerospace Engineering, Princeton University, Princeton, New Jersey, United States of America; 3 Department of Psychology, University of Alberta, Edmonton, Alberta, Canada; French National Centre for Scientific Research, France

## Abstract

When faced with risky decisions, people tend to be risk averse for gains and risk seeking for losses (the *reflection effect*). Studies examining this risk-sensitive decision making, however, typically ask people directly what they would do in hypothetical choice scenarios. A recent flurry of studies has shown that when these risky decisions include rare outcomes, people make different choices for explicitly described probabilities than for experienced probabilistic outcomes. Specifically, rare outcomes are overweighted when described and underweighted when experienced. In two experiments, we examined risk-sensitive decision making when the risky option had two equally probable (50%) outcomes. For experience-based decisions, there was a reversal of the reflection effect with greater risk seeking for gains than for losses, as compared to description-based decisions. This fundamental difference in experienced and described choices cannot be explained by the weighting of rare events and suggests a separate subjective utility curve for experience.

## Introduction

How people evaluate risk and decide between risky alternatives is a fundamental problem in decision making—one that should perhaps take on renewed importance in light of the recent financial crisis [1]–[3]. Risk sensitivity in humans is most commonly evaluated by asking people to decide between explicitly described, hypothetical choice scenarios [4]–[5]. Yet frequently in life, we make repeated choices, and our knowledge about uncertain outcomes is gleaned from experience, rather than from explicitly described scenarios. Recently, researchers have begun to evaluate risky choice based on experience, and several studies have reported that experience-based choices may differ from choices based on verbal descriptions. In particular, a *description-experience gap* has been revealed in people's sensitivity to rare outcomes [5]–[14]. When making decisions based on verbal descriptions, people overweight rare events, but they underweight those rare events when making decisions based on experience. In this paper, we show how this difference between decisions from description and experience extends beyond rare events, such as the proverbial black swan, and occurs even for events that are equally probable, such as tossing a coin.

When given a choice between explicitly described options, people tend to be risk averse for gains and risk seeking for losses. For example, when presented with a choice between a guaranteed win of $100 or a 50/50 chance of winning $200, most people will select the sure win and take the $100. Alternatively, if given a choice between a sure *loss* of $100 or a 50/50 chance of losing $200, most people will gamble and take the risky option. This shift from risk aversion for gains to risk seeking for losses is known as the *reflection effect* and is a foundational result in behavioral economics [4], [15]–[16]. This reflection effect is often interpreted, within the guise of prospect theory, as reflecting an s-shaped utility curve, whereby the subjective utility of gaining $200 is less than twice as good as the subjective utility of gaining $100, and the subjective utility of losing $200 is less than twice as bad as the subjective utility of losing $100. As a result, people tend to avoid the gamble in the gain case, but seek out the gamble in the loss case. This asymmetry can arise even when the objective expected value of both options in both choice settings is identical [4], [16]–[19].

If, however, one of the two outcomes is comparatively “rare”, usually defined as less than 20% chance of occurrence, then a different pattern of results emerges when people are asked to decide based on their experiences [6], [11]. For example, Hertwig et al. [10] (Problems 1 and 4) presented people with a choice between 100% chance at gaining $3 or an 80%/20% chance at gaining $4/$0. The description group received a verbal description of the contingencies. The experience group was allowed to repeatedly sample from the different alternatives and get feedback before making a single rewarded choice. They found that the experience group chose the risky alternative much more often than the description group (i.e., the experience group was more risk seeking). In contrast, when the choice was changed to be between losses, but keeping the amounts and probabilities the same, the experience group chose the risky alternative much less often the description group (i.e., the experience group was more risk averse). These results are commonly interpreted as an underweighting of the rare outcome in the experience-based decision process, possibly due to estimation error or a recency bias [11]. These results, however, hint at something even more fundamental: Perhaps the entire s-shaped curve mapping from objective value to subjective utility is altered when people learn about risky contingencies from experience. As a result, differences between description- and experience-based decisions should appear even in the absence of rare events. To evaluate this possibility, we designed two experiments that examine decisions from experiences without any rare outcomes, where the risky option always led to one of two equiprobable (50%) outcomes.

**Figure 1:**
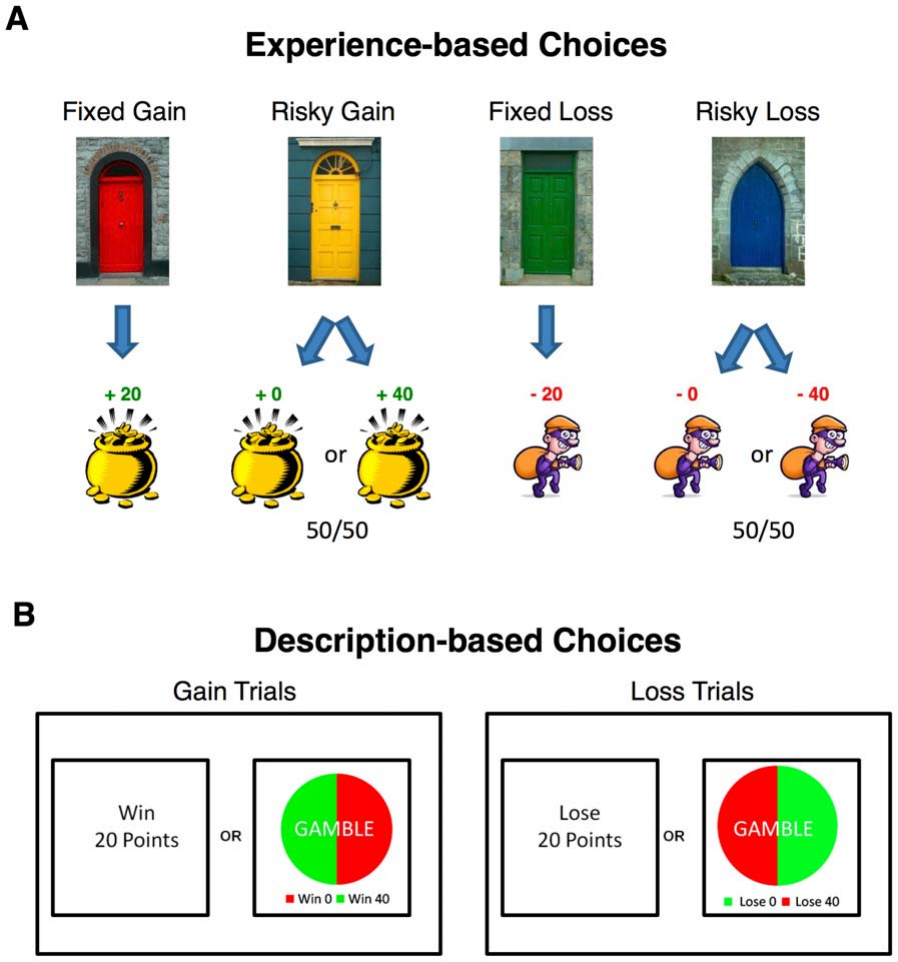
Schematic of the method used in the experiments. (A) On *experience*-based choice trials, participants were faced with a choice between two of four possible colored doors. Two doors always led to losses, and the other two always led to gains. Their choice was immediately followed by a gain or loss of a fixed or variable number of points throughout Experiment 1 and during the pre-training trials only in Experiment 2. (B) On *description*-based choice trials, participants were presented with verbal and pictorial descriptions of different choices between fixed or variable number of points. No feedback was given until all trials in a run were complete to prevent participants from learning from their experience.

In this pair of experiments, we demonstrate that the differences between described and experienced risky choices are not limited to rare events. We developed a novel task for decisions from experience and description, wherein the same participants were repeatedly tested for risky choice in both ways (Fig. 1). In the *experience* conditions, participants chose between two colored doors and then immediately gained or lost points. One door led to a guaranteed win of 20 points, whereas a second door was followed by a 50/50 chance of winning 0 or 40 points. The final two doors used the same contingencies, but were followed by losses instead of gains. In the *description* conditions, the same participants chose between losing (or winning) a guaranteed number of points and a gamble where they could lose (or win) twice as many points. No immediate feedback was given on the outcome of this described gamble. Based on traditional prospect theory, we would expect risk aversion for gains and risk seeking for losses in both conditions. If, however, decision making from experience does not conform to prospect theory, even in the absence of rare events, then we would expect a difference between the experience and description conditions.

In this first experiment, experience-based choices always produced immediate feedback, and described choices never resulted in immediate feedback, perhaps leading to differential emotional states during the experienced and described choices. A second experiment controlled for the possibility that participants may have been in a more emotionally aroused or “hot” state during the experience-based choices. Risky decision making has been shown to vary in emotional states [23]–[24], suggesting that perhaps any differences between experience and description may arise from differential engagement of emotional and cognitive systems [25]. The second experiment was designed to eliminate the possibility that participants were in a different “hot” state during the experienced trials. The experiment started with a separate training period during which participants learned the contingencies between the doors and potential outcomes. All subsequent testing for both experienced and described trials was conducted without any further feedback. In addition, all test runs contained both a block of experienced trials and a block of described trials. These two changes should help ensure similar emotional states during the test period.

## Methods

### Experiment 1: Partial Feedback

#### Participants

A total of 62 participants were divided into 2 groups: 34 in Group 1 and 28 in Group 2. All participants were University of Alberta undergraduate students, who participated for course credit. Participants were tested in squads of 2–4 people. Written informed consent was obtained from all participants after the objectives and risks of the study were explained, and all procedures were approved by the Arts, Science, & Law Research Ethics Board (ASL REB) at the University of Alberta. Data from 6 participants were removed due to poor performance on catch trials (see Results for details). The remaining participants consisted of 35 females and 21 males, with a mean age of 20 (range of 18 to 39).

#### Procedure

Figure 1 schematically depicts the procedure on the two types of trials in the experiment. On the experienced trials, participants had to click a door, which was immediately followed by feedback. There were 4 different-coloured doors in the experiment. On some trials there were 2 doors to choose from (choice trials), and, on other trials, there was only 1 door that had to be selected to continue (single-option trials). The feedback lasted 1200 ms and consisted of the number of points gained or lost along with a little graphic; for gains, a pot of gold was displayed, and for losses, a robber was displayed. On the bottom of the screen, a running tally of the points earned thus far was displayed. Each door led to a different outcome: a fixed gain (+20), risky gain (+0 or +40 with 50% probability), fixed loss (−20), or risky loss (−0 or −40 with 50% probability).

Experienced trials were presented in 3 runs of 56 trials that were each divided among 3 basic types: 32 choice trials between the two different gains or the two different losses (16 of each), 16 catch trials with one gain option and one loss option, and 8 single-option trials where only one door was presented and had to be selected. The ordering of these trials within a run was randomized for each run. These numbers were selected so that each door would appear equally often on both sides of the screen and in combination with the other doors. Both the 32 regular choice trials and the 8 single-option trials in each run were equally divided between gain trials and loss trials, and thus had an expected value of 0. The 16 catch trials were the only trials on which participants could earn a net gain of points and served to ensure that participants paid attention to their choices.

An additional block of 24 single-option trials (16 gain and 8 loss) immediately preceded the very first run. These extra single-option trials ensured that the participants initially encountered exactly the planned distribution of outcomes for the different doors. In these single-option trials, each of the 4 doors appeared 4–8 times in a random order. For the risky doors, the outcomes consisted of exactly half wins and half losses on those first few trials, ensured by random selection (without replacement) from a pool of outcomes for each door. As a result, there was a guaranteed 50/50 distribution over the first few exposures to the door. The order was randomly determined for each participant. Note the fixed number of choice and single-option trials: Participants did not get to choose how many samples they encountered before making a decision, in contrast to the common procedure for examining experience-based decision making [10]. The two groups of participants differed primarily in that door color was counterbalanced across groups.

On the described trials, two options were presented on the screen separated by the word “or”. One side of the screen showed a fixed option and presented either the words “Win 20” or the words “Lose 20”. The other side of the screen showed a risky option and presented a pie chart with the word “Gamble” written in the middle. The pie chart was half red and half green (see Figure 1). For the risky loss option, the two halves of the pie chart corresponded to “Lose 40” (red) and “Lose 0” (green). For the risky gain option, the two halves of the pie chart corresponded to “Win 40” (green) and “Win 0” (red). The design of the described trials was inspired by the method of [20]. The described trials were presented in 2 runs of 48 trials divided among 2 basic types: 32 choice trials between the two different gains or the two different losses (16 of each) and 16 catch trials with one gain option and one loss option. For Group 2, there were 20 catch trials, including ones between two gains or losses of different objective values (e.g., “Win 10” vs. 50/50 chance of “Win 40” or “Win 0”). Participants were advised in advance that no feedback would be given during these runs, and the running tally did not appear on the bottom of the screen.

The experimental session was divided into 5 runs that lasted about 6–7 minutes each. The first, third, and fifth runs consisted exclusively of experience-based decisions, whereas the second and fourth runs consisted exclusively of description-based decisions. Due to a computer error, 3 participants in Group 1 missed the first experience run and only received the final two experience runs. Runs were separated by a riddle for entertainment and a brief rest period. To enhance motivation to perform the task, a list of anonymous high scores was posted on the blackboard, and participants were encouraged to try to beat the scores. All trials were counterbalanced for side so that each option appeared equally often on either side of the screen. An inter-trial interval of 2.5–3.5 s separated all trials. Stimuli were presented and the data recorded with E-prime 1.0 (Psychology Software Tools, Inc.; Pittsburgh, PA).

#### Data Analysis

Two primary dependent measures were used. To compare performance for gains and losses, gambling quotients were calculated as the probability of choosing the risky option. Only data from the final run were used to compare the described and experienced cases to exclude any potential changes across runs due to learning. A preliminary three-way ANOVA on gambling quotients with group as a between-subjects factor indicated no effect of group, nor any significant interactions, and thus the two groups were collapsed for all analyses. Gambling quotients were then compared using a two-way (condition and choice type), repeated-measures ANOVA, followed by pairwise comparisons. Corrections for multiple pairwise comparisons were performed with the Holm-Sidak iterative method. To measure the strength of the reflection effect, we calculated reflection scores that were the difference between the gambling quotients for losses and gains. These reflection scores were compared across description and experience using a paired t-test. Effect sizes were calculated as Cohen's *d* for *t* tests and partial eta-squared (*η* ^2^ *_p_*) for ANOVAs [21]–[22]. Inferential statistics were calculated using SigmaPlot 11 (Systat Software, Inc.; San Jose, CA), SPSS 18 (SPSS, Inc.; Chicago, IL), and MATLAB (The Mathworks Inc.; Natick, MA).

### Experiment 2: No Feedback

#### Participants

Twenty-eight new participants from the same student population were run in Experiment 2. One participant scored less than 60% on the experienced catch trials; data from this participant have been removed from all analyses. The remaining participants consisted of 24 females and 3 males, with a mean age of 24 (range of 18 to 44).

#### Procedure

The basic procedure on each trial was identical to Experiment 1. The biggest difference from Experiment 1 was that the experienced trials were now divided into a pre-training period with feedback and a test period with no feedback. The pre-training period began with 32 single-option trials (8 with each door) and then an additional 104 trials (32 single option, 48 choice, and 24 catch trials in a random order), all followed immediately by rewarding feedback. After a riddle and a brief break, participants were then exposed to 8 sample described trials to familiarize them with the procedure. There were then 6 additional runs that mixed experienced and described trials, all with no feedback. Each run had one block of 24 described trials (33% gain, 33% loss, 33% catch) and another block of 25 experienced trials (40% gain, 40% loss, 20% catch), presented in a counterbalanced order across runs. Unlike Experiment 1, the running total on the bottom of the screen and tally of high scores on the chalkboard in the room were not present. At the end of the experiment, subjects were presented with a questionnaire that asked them what they had learned about the different doors. Data analysis proceeded as in Experiment 1, except all 6 non-feedback runs were used to compare the described and experienced trials.

## Results

### Experiment 1: Partial Feedback

**Figure 2:**
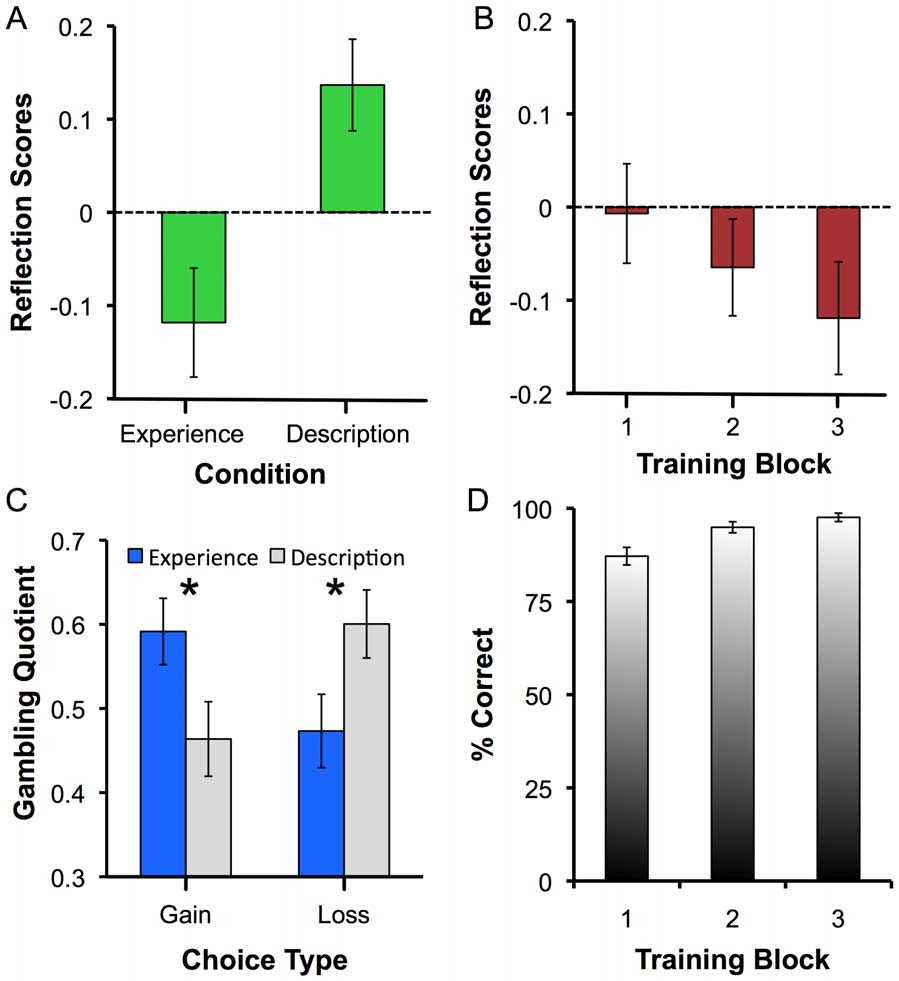
Results from Experiment 1. (A). Reflection scores as a function of experimental condition. Participants displayed reliably higher reflection scores for described vs. experienced problems (*p*<.001). (B). Reflection scores on experienced trials as a function of block in the experiment. There was a trend toward a greater reverse reflection effect as the potential outcomes were learned. (C). Gambling quotient as a function of experimental condition and choice type. For gains, participants were risk seeking for experienced problems, but risk averse for described problems. In contrast, for losses, participants were risk averse in experienced problems, but risk seeking in described problems. * =  *p*<.05. (D). Percentage correct on catch trials as a function of experience training block. Performance increased across blocks, but was high throughout, peaking at 97.6% on the final training block.

Contrary to the prediction of prospect theory, we found a reversal of the reflection effect for the experienced problems, even with equiprobable outcomes in Experiment 1. Figure 2A shows how people gambled more for gains than losses when the outcomes were learned from experience, but not when they were described. The reflection scores, calculated as the probability of gambling on loss trials minus the probability of gambling on gain trials, were significantly higher on described trials than on experienced trials (*t*(55) = 4.45, *p*<.001, *d* = .60). The reflection scores for experience-based decisions trended downward across trials (see Fig. 2B), indicating that this reversal became more established as the relationships between cues and rewards were learned more accurately, although the visible downward trend was not statistically reliable (*F*(2,106)  = 1.98, *p* = .14, *η* ^2^ *_p_* = .04). Figure 2C shows that this difference between the experienced and described trials represents a full reversal of the reflection effect (condition × choice type interaction, *F*(1,55)  = 19.9, *p*<.001, *η* ^2^ *_p_* = .27). On gain trials, participants flipped from risk aversion on described problems to risk seeking on experienced problems. On loss trials, participants flipped from risk seeking on described problems to risk aversion on experienced problems (*p*<.031 for all pairwise comparisons).

Figure 2D depicts mean performance on the experienced catch trials across the three experience runs of the experiment. Six participants scored less than 60% correct on these catch trials (4 in Group 1 and 2 in Group 2); data from these participants have been removed from all analyses. For the remaining 54 participants that received all 3 training runs, performance was high throughout, but improved slightly across the runs (*F*(2,106)  = 14.99, *p*<.01, *η* ^2^ *_p_* = .22). In the final run, on which the primary comparisons between described and experienced trials are based, mean performance on catch trials was 97.6% correct. Across the whole experiment, the empirical probabilities of receiving the better outcome on the risky option were 51.6±.8% and 50.3±1.2% for gain and loss trials respectively, which were not statistically different than 50%, nor from each other (all *p*s>.05).

### Experiment 2: No Feedback

**Figure 3:**
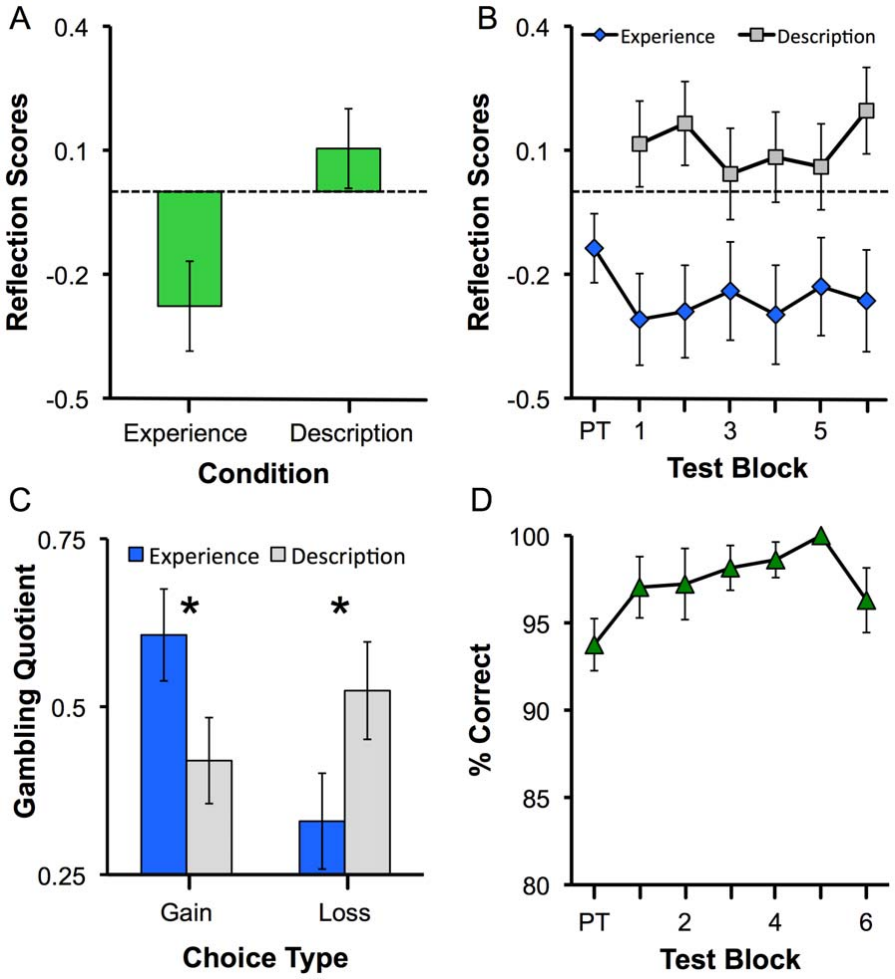
Results from Experiment 2. (A). Reflection scores as a function of experimental condition. Participants displayed reliably higher reflection scores for described vs. experienced problems (*p*<.001). (B). Reflection scores on experienced and described trials as a function of block in the experiment. On each block, reflection scores were higher for described than for experienced trials. PT  =  pre-training for the experienced trials, where feedback was obtained after each trial. (C). Gambling quotient as a function of experimental condition and choice type. For gains, participants were risk seeking for experienced problems, but risk averse for described problems. In contrast, for losses, participants were risk averse in experienced problems, but nominally risk seeking in described problems. * =  *p*<.05. (D). Percentage correct on catch trials as a function of experience training block. Performance was relatively stable across test blocks, peaking at 100% on test block 5.

As in Experiment 1, people again gambled more for gains than losses when these contingencies were learned from experience, but not when they were described, even when immediate feedback was no longer being provided about the experience-based choices. Figure 3A depicts how the reflection scores were significantly lower in the experienced trials, (*t*(26)  = 2.79, *p*<.01, *d* = .54). These reflection scores were consistent across the 6 non-feedback runs for both experienced and described trials, as shown in Figure 3B. A two-way ANOVA confirmed a main effect of trial type (*F*(1,27)  = 7.83; *p*<.01, *η* ^2^ *_p_* = .23), but no effect of run, nor any interaction (both *p*s>.25). The difference between experienced and described trials was present for both gains and losses. As depicted in Figure 3C, when choosing between gains, participants gambled more in the experienced case, and when choosing between losses, participants gambled more in the described case (condition × choice type interaction, *F*(1,26)  = 7.82; *p* = .01, *η* ^2^ *_p_* = .23; both pairwise comparisons, *p*<.038). In addition, there was a significant reversal of the reflection effect for the experienced trials (*p*<.01); however, the reflection effect for the described condition failed to reach significance (*p*>.05). Finally, performance on the catch trials was high throughout, even peaking at 100% correct on the 5^th^ run (see Fig. 3D).

## Discussion

In a pair of experiments, we show that the classic reflection effect from behavioral economics is reversed when participants learn the reward contingencies from experience. These results significantly extend the recent discovery of a description-experience gap in the assessment of rare events [5]–[14]. For the variable options in our experiments, both outcomes were equiprobable, meaning that neither event was more rare than the other. Despite this elimination of rare events from the experiment, we still found a profound difference between people's risky choices in described and experienced cases. This finding suggests that the description-experience gap is more general than previously thought and cannot be solely explained by an overweighting of rare events [11]. Moreover, by using a within-subject design, our study is one of few studies to show differences between experience and description in the very same participants [7]–[13]. Our novel finding demonstrates a serious limitation to current theories of risky choice in behavioral economics, which are mostly based on described choices [4]–[5].

In prospect theory, the subjective utility curve grows sub-linearly, leaving extreme values proportionally underweighted [4], [16]. That is, a reward that is objectively twice as large (e.g., $200 vs. $100) is perceived as subjectively less than twice as big. In contrast, our results suggest that the subjective utility curve based on experienced outcomes may overweight extreme values, growing perhaps supra-linearly (i.e., faster than linear). Thus, extreme values (big wins or big losses) carry proportionally more weight in decisions based on experience, leading to risk seeking for gains and risk aversion for losses. This *extrema* hypothesis supposes that, in the experienced case, the largest and smallest rewards in a given context are given undue importance in the decision-making process. One possibility is that people remember the big wins and the big losses best, and, as a result, their decision making is swayed by those extreme outcomes, perhaps through an affect [26] or availability heuristic [27]. In our experiments, this memory bias towards extreme values would result in gambling for gains and risk aversion for losses, but only when the outcomes were learned from experience (as seen in Figs. 2 and 3).

The somatic marker hypothesis also provides a potential mechanism for this weighting scheme that gives a disproportionately large weight to extreme values [28]–[29]. This hypothesis contends that emotional, bodily reactions to rewarding events drive subsequent decision making. Larger rewards (good or bad) would thus elicit relatively more potent and memorable emotional responses, driving subsequent decision-making, but only in the experienced, retrospective case. We did not, however, record any physiological measures as a proxy for emotional reactions and thus cannot definitely claim that the extreme values were indeed more emotionally salient. An extended range of values beyond those presented here would also help formulate a hypothesis as to exactly what function might describe this experience-based weighting.

In a recent review, Hertwig and Erev [11] proposed several psychological mechanisms that might account for the description-experience gap for rare events. Our finding that this gap can extend to equally probable outcomes suggests that some of these mechanisms are insufficient for explaining the differences between description and experience. For example, our results cannot easily be accounted for by limited sample sizes or estimation errors of the rate of occurrence of rare events. Indeed, the programmed and received outcomes for the risky outcomes always hovered near 50% in our experiment. In addition, the single-option trials ensured that all participants received both possible outcomes several times for both risky options, and many of our participants correctly identified the exact 50/50 probability in the post-experiment questionnaire. As compared to many other studies of decisions from experience [10], [13], our participants received more experience with the different options (>100 trials total as opposed to the usual 10–20) further limiting the possibility that a sampling bias could explain the results. This extensive training, however, raises a different concern in that our results only seemed to emerge after significant training with the experience-based options (see Fig. 2B). We do not know yet whether our results will generalize to other procedures for examining experience-based decisions that rely on fewer exposures to the potential outcomes.

A second possibility that Hertwig and Erev [11] suggest for explaining the description-experience gap with rare events is that recent events might carry more weight in the decision process, again biasing the weight toward the more frequently occurring outcome. In our experiments, however, there were no rare events, therefore neither the positive nor the negative outcome for the risky option should have received consistently increased weighting. Moreover, the results from Experiment 2, which explicitly separated the learning phase with feedback from the experience-based test trials, provide further evidence against this recency hypothesis.

Another possible difference between the described and experienced problems is that the probabilities of the various outcomes are known with certainty in the described problems, but start off uncertain or ambiguous for the experienced problems [30]–[31]. Thus, to the extent that participants have not learned the relationship between the stimuli and rewards, the experienced task incorporates elements of ambiguity. The trend towards increasingly negative reflection scores across the different blocks in Exp. 1 (Fig. 2D), however, suggests that ambiguity does not underlie the reversed reflection effect. As the contingencies are learned (see Fig. 2B) and the ambiguity in the experienced problems is attenuated, the difference between the experienced and described problems does not disappear. Indeed, the difference between experienced and described problems is most robust on the final experience run, after the contingencies are well learned (Fig. 2A and 2C).

Our findings suggest an alternate interpretation of the role of ambiguity in other tasks that involve ambiguous outcome probabilities, such as the Iowa Gambling Task. The Iowa Gambling task engages emotional processes to a greater extent than tasks with stated probabilities [28]–[29], [32]–[33]. This enhanced emotional engagement has been attributed to ambiguous decisions in those tasks. We suggest that the emotional engagement might instead derive from the fact that the outcomes for those ambiguous decisions are learned from experience.

Our results strongly reinforce the finding that patterns of human decision-making under uncertainty depend on how the decision problem is posed, as is often found in many areas of psychology [19], [34], [35], [36]. The canonical reflection effect can be reversed when participants learn the probabilities from experienced outcomes, even for moderate probabilities, indicating that how we decide may be fundamentally different when we think about the future (in described cases) than when we reflect on the past (in experienced cases). When considering future possibilities, we may underweight extreme outcomes, as per prospect theory, leading to the canonical reflection effect. When remembering past outcomes, we may be driven by the emotional, somatic effects of the more extreme values and overweight those outcomes in our decision making for risky outcomes, leading to a reversal of the usual reflection effect. This dichotomy between prospective and retrospective modes of evaluation suggests a fundamental extension to theories of risky choice.
